# Biosynthesis of gamma-aminobutyric acid by *Lactiplantibacillus plantarum K16* as an alternative to revalue agri-food by-products

**DOI:** 10.1038/s41598-022-22875-w

**Published:** 2022-11-07

**Authors:** Lucía Diez-Gutiérrez, Leire San Vicente, Jessica Sáenz, Argitxu Esquivel, Luis Javier R. Barron, María Chávarri

**Affiliations:** 1 TECNALIA, Basque Research and Technology Alliance (BRTA), Health and Food Area, Health Division, Parque Tecnológico de Álava, Leonardo Da Vinci 11, 01510 Miñano, Spain; 2 Lactiker Research Group, Faculty of Pharmacy, University of the Basque Country (UPV/EHU), Paseo de La Universidad 7, 01006 Vitoria-Gasteiz, Spain

**Keywords:** Applied microbiology, Industrial microbiology, Bacterial secretion

## Abstract

Probiotic metabolites, known as postbiotics, have received attention due to their wide variety of promoting health effects. One of the most exciting postbiotic is gamma-aminobutyric acid (GABA), widely produced by lactic acid bacteria, due to its benefits in health. In addition, the performance of the biosynthesis of GABA by *Lactiplantibacillus plantarum* could be modulated through the modification of fermentation parameters. Due to their high nutritional value, agri-food by-products could be considered a useful fermentation source for microorganisms. Therefore, these by-products were proposed as fermentation substrates to produce GABA in this study. Previously, several experiments in Man Rogosa Sharpe (MRS) broth were performed to identify the most critical parameters to produce GABA using the strain *Lactiplantibacillus plantarum K16*. The percentage of inoculum, the initial pH, and the concentration of nutrients, such as monosodium glutamate or glucose, significantly affected the biosynthetic pathway of GABA. The highest GABA yield was obtained with 500 mM of monosodium glutamate and 25 g/L of glucose, and an initial pH of 5.5 and 1.2% inoculum. Furthermore, these investigated parameters were used to evaluate the possibility of using tomato, green pepper, apple, or orange by-products to get GABA-enriched fermented media, which is an excellent way to revalorise them.

## Introduction

Probiotic microorganisms are now widely consumed worldwide due to their potential to preserve and enhance human health^1^ through their direct effect on the intestinal microbiota, modulation of the immune system, protection against pathogens colonisation, or reduction of oxidative stress, among others^2^. These health benefits can be produced because of the positive interaction between probiotics and the host gut microbiota, triggering the activation of different intracellular signalling pathways^3^. For example, the activation of genes involved in the synthesis of mucin avoids pathogens' adhesion to the gut barrier, the enhancement of phagocytosis through the increase of macrophages or the attenuation of pro-inflammatory cytokines production^4^.

Likewise, probiotics can also produce host benefits by metabolising different nutrients and producing bioactive compounds classified as postbiotics which can be defined as metabolites synthesised by these microorganisms or other compounds released during fermentation processes^5–9^. A wide range of compounds could be classified as postbiotics, such as vitamins, minerals, amino acids, neurotransmitters, or lipid compounds^10^. One of the most promising postbiotic is the neurotransmitter gamma-aminobutyric acid (GABA)^11,12^. This compound can reduce anxiety and depression in humans, influence several neurochemical pathways, enhance the immune system, or modulate blood pressure decreasing the likelihood of developing heart problems^13^.

Consistent with the health benefits of GABA, this compound was initially produced industrially by chemical synthesis to meet pharmaceutical and food companies’ demands^14^. However, the poor synthesis performance, the detrimental effect on the environment, and the low profitability of the process led to the substitution of the chemical production with a more suitable production by using a biotechnological process carried out by microorganisms^15^. Some examples of interesting GABA producers are lactic acid bacteria (LAB)^16^, *Bacillus subtilis*^17^, *Aspergillus oryzae*^18^, *Listeria monocytogenes*^19^ *or Bifidobacterium*^20^. Among these microorganisms, LABs have been considered one of the most attractive alternatives to synthesise GABA due to the high performance of their biosynthetic process, their classification as generally regarded as safe (GRAS) microorganisms and their potential beneficial effects on human health^21^.

The synthesis of GABA is commonly performed through the glutamic acid decarboxylase pathway (GAD) as a mechanism triggered under stressful environments. Specifically, a molecule of l-glutamic acid (l-Glu) is decarboxylase by a GAD enzyme resulting in the production of a GABA molecule^22^. Usually, the GAD enzyme is encoded by a *gadB* gene, but some LAB can present two genes such as *Levilactobacillus brevis* which has a *gadA* and *gadB* gene. Moreover, some species, such as *Lactobacillus buchneri, Lb. curvatus or Lb. sakei,* could even present potential transcriptional regulators that can enhance the synthesis of GABA^21^. For instance, Gong et al.^23^ highlighted how the transcriptional regulator GadR presented in *L. brevis* is directly linked to the high GABA yield and the resistance against acid environments of this bacteria. Due to the diversity of genetics involved in the GAD system, the GABA yield could be very different between species such as, *L. buchneri WPZ001* yielded 117 g/L of GABA^24^, *L. brevis NCL912* produced 103.7 g/L of GABA^25^ or *Lactiplantibacillus plantarum (Lactobacillus plantarum) N5* yielded 21.8 g/L of GABA^26^. Cui et al.^21^ explained that the GAD systems is strain-specific and even strains with the same GAD system could have different yield of GABA.

Within LAB, *L. plantarum* strains could produce a great amount of GABA, depending on the source where they were isolated from, and the yield of the machinery involved in the biosynthetic pathway of GABA^27^.

GAD pathway performance can be modulated by adjusting several environmental parameters such as temperature, initial pH, or oxygen availability. In addition, the type and concentration of minerals, coenzymes, nitrogen, carbon sources, and other additives could positively influence GABA biosynthesis^28^. Several studies have been conducted to adjust the main physic-chemical parameters involved in the GABA synthesis. For instance, Sharafi and Nateghi^29^ optimised the GABA production by *L. brevis* by studying the effect of temperature, initial pH, l-Glu, concentration and fermentation time. They obtained that the fermentation carried out at 34 °C, with an initial pH of 4.65, 650 mmol of l-Glu and for 96 h of incubation time, enhanced more than twice the synthesis of GABA compared to non-optimised conditions. Wu et al.^30^ and Song and Yu^31^ reported that the inoculum percentage and the nitrogen and carbon source type could positively influence the GABA synthesis by *Lactobacillus* strains.

Furthermore, these optimisation processes are generally performed using MRS broth, characterised by the high concentration of nutrients necessary for *Lactobacillus* growth. However, the wide variety of nutrients used in this culture media increases the cost of the production process. Thus, it is not considered a suitable fermentation media for scale-up production^32^. During the last years, by-products from the agri-food industry has gained attention to be used as low-cost fermentation media which puts a value on potential pollutants.

In general, agri-food industries generate a considerable amount of waste mainly produced from the transformation of raw fruits and vegetables into final products like juices or smoothies, which normally discard structural parts such as seeds, peels, leaves, or pulps. Mnisi et al.^33^ reported that from 900 million metric tons of fruit production in 2020, approximately a 30% was discarded, normally producing a strong environmental impact because these by-products are normally burned or placed in landfills^34^, although it is also being used to produce animal feed^35^. Consequently, the use of these agri-food by-products as culture media for fermentation processes can be a good way to revalorise this type of waste, as well as to produce bioactive compounds useful for the formulation of new drugs and functional foods^36^.

Falah et al.^37^ proposed to use molasses, dairy sludge, and soybean meal as fermentation media to produce GABA by *L. brevis, Limosilactobacillus fermentum* and *L. plantarum.* Zarei et al.^38^ made a functional drink using whey protein, considered a high environmental impact waste product, as the primary source to synthesise GABA by *L. plantarum*. In our previous study, *L. plantarum K16* was isolated from Kimchi and identified as GABA-producer. Then, it was evaluated how parameters such as temperature, the concentration of yeast extract and incubation time influenced the GABA production by *L. plantarum K16*^39^ Therefore, the objective of this study was to continue with the analysis of parameters, such as inoculum percentage, initial pH, monosodium glutamate (MSG) concentration, and glucose concentrations, involved in the GABA production of *L. plantarum K16* using MRS broth and achieve the highest yield of GABA in this medium. Afterwards, a fermentation trial was performed to determine if tomato, green pepper, apple, or orange by-products could be considered as suitable fermentation substrates to obtain GABA-rich fermented products.

## Methods

### Microbial strain

LABs were isolated from kimchi through standard culturing methods in the Food Biotechnology laboratory (TECNALIA, Miñano, Spain). The isolated LABs were grown in MRS broth supplied with l-Glu, and the supernatants were collected to analyse the GABA content using ultra-high-performance liquid chromatography (UHPLC) coupled with mass spectrometry (MS). Only one of the isolated LABs was able to produce GABA which was identified as *L. plantarum K16.* Therefore, *L. plantarum K16* was used to evaluate how different parameters could modulate the synthesis of GABA.

### GABA production by *L. plantarum* K16 strain

The optimisation process for GABA synthesis by *L. plantarum K16* strain was carried out in several stages following a one-factor-at-a-time (OFAT) experimental design in MRS broth (Sigma-Aldrich, Madrid, Spain). Therefore, the optimisation process was performed by studying different levels of one fermentation parameter while keeping unchanged the other fermentation parameters. The beginning of this optimisation process was explained in a previous study^39^, where the incubation temperature, concentration of yeast extract and fermentation time were evaluated. The results of this experiments indicated that the initial conditions to continue the optimisation process should be MRS broth supplied with 5 g/L of glucose, 12 g/L of yeast extract, an initial pH of 5.5, inoculum of 1%, 500 mM of MSG, incubation temperature of 34 °C and 96 h of fermentation. Furthermore, in the current research the fermentation parameters studied in MRS broth were inoculum percentage, initial pH, MSG concentration and glucose concentrations. For each experiment, an inoculum of *L. plantarum K16* was prepared in MRS broth overnight at 37 °C. Then, the amount of GABA production (mg/L; ± 0.01) was quantified by UHPLC-MS. Likewise, the microbial growth was measured by plating serial dilutions in MRS agar and counting colonies to calculate the colony-forming units (CFU) and expressed as log CFU/mL (± 0.01). Finally, the pH value of the fermented medium was measured (± 0.1) with a Crison Basic 20 pHmeter (Crison, Barcelona, Spain).

### Inoculum percentage

According to the research of Kantachote et al.^40^, different percentages of *L. plantarum K16* strain were used in the fermentation process. The fermentation media was prepared, with 250 mL Erlenmeyer flask containing 100 mL of working volume, by adding 5 g/L of glucose to MRS broth composed by 12 g/L of yeast extract, adjusted to an initial pH of 5.5 and sterilised by autoclaving the culture medium at 121 °C for 15 min. Afterwards, the medium was enriched with 500 mM of sterilised MSG, further inoculated with 0.8, 1.0, 1.2 and 1.4% of *L. plantarum K16* strain and incubated at 34 °C without shaking. After 96 h of fermentation, analytical samples of the fermented medium were taken to determine the pH, GABA amount and the CFU/mL.

### Initial pH

The MRS broth was prepared as previously described for glucose, yeast extract and MSG concentrations, and 4.0, 4.5, 5.5 and 6.0 as different initial pH values. An inoculum percentage of 1.2% was selected as the optimum value obtained for GABA production from the previous stage, and the fermentation medium was incubated at 34 °C during 96 h. Likewise, analytical samples of the fermented medium were taken to determine the pH, GABA amount and the CFU/mL.

### MSG concentration

Different MSG concentrations (100, 300, 500 and 550 mM) were evaluated to determine how this precursor of the GAD biosynthetic pathway could influence the production of GABA. The fermentation media was prepared by adding 5 g/L of glucose to MRS broth composed by 12 g/L of yeast extract. Following the results obtained in the previous OFAT stages, the initial pH was fixed to 5.5, the medium was inoculated with 1.2% of *L. plantarum K16* strain, and the medium was incubated at 34 °C during 96 h. Also, analytical samples of the fermented medium were taken to determine the pH, GABA amount and the CFU/mL.

### Glucose concentration

According to the scientific literature, glucose was chosen as the best carbon source for the optimisation of GABA production by LAB fermentation^30,41^. Different glucose concentrations (20, 23, 25 and 27 g/L) were tested in the MRS broth containing 12 g/L of yeast extract and 500 mM of MSG. This MSG concentration was selected as the optimum value obtained for GABA production from the previous section. As other assays, initial pH was adjusted to 5.5, the medium was inoculated with 1.2% of *L. plantarum K16* strain and incubated at 34 °C for 96 h. Moreover, analytical samples of the fermented medium were taken to determine the pH, GABA amount and the CFU/mL.

### GABA production using agri-food by-products

The previous studies carried out in MRS broth helped to evaluate how different fermentation parameters could influence and improve the production of GABA by *L. plantarum K16.* Thereafter, different agri-food by-products such as tomato, green pepper, apple, and orange pulp and seeds (obtained from private suppliers) were selected to be used as fermentation substrates to produce GABA (these agri-food by-products were obtained and treated following general guidelines and legislation for experiments carry out with plants). Table 1 shows the main nutritional composition such as carbohydrates, total sugars, protein, fat, amino acids, or minerals of the tomato, green pepper, orange, and apple obtained from European Food Information Resource^42^. The fermentation media from agri-food by-products were firstly prepared by grinding and re-suspending independently 5 g of each by-product into distilled water by stirring. Due to the importance of glucose and yeast extract in *L. plantarum K16* to produce GABA, the media was enriched with extra 25 g/L of glucose and 12 g/L of yeast extract. Subsequently, the pH was adjusted to 5.5 and the medium sterilised by autoclaving^43^. After the sterilisation, these agri-food by-product media were supplied with 500 mM of the precursor MSG, inoculated with 1.2% of *L. plantarum K16* strain and incubated at 34 °C during 96 h. As before, analytical samples of the fermented medium were taken to determine the pH, GABA amount and the CFU/mL.

**Table 1:** Nutritional composition (mg/100 g) of tomato, apple, orange, and green pepper by-products^35^.

Composition	Tomato	Apple	Orange	Green pepper
Total carbohydrates	3990	11,400	8900	1600
Total sugars	3350	10,350	8880	1530
Total fat	190	360	200	800
Total protein	950	310	870	630
**Amino acids**				
Alanine	16	11	25	25
Aspartic acid	103	7	99	89
Arginine	13	6	63	30
Proline	19	6	17	27
Isoleucine	25	6	17	20
Leucine	12	13	28	32
Valine	18	12	29	26
Glutamic acid	335	25	57	82
**Minerals**				
Calcium	12	6	41	11
Magnesium	11	6	15	10
Potassium	248	120	165	120
Sodium	4	1	1	4
Phosphorus	33	9	5	Nd
Iron	0.2	0.6	0.5	0.5
Selenium	Traces	Traces	Traces	1
Zinc	0.1	0.1	0.2	0.1

### GABA analysis by UHPLC-MS

An ACQUITY UPLC H-class system (Waters., Milford, MA, USA) with a HILIC column (130 Å pore size; 1.7 µm particle size; 2.1 mm internal diameter; 100 mm length) (Waters) coupled with a SecurityGuard ULTRA Cartridge pre-column (Waters) was used for the analysis of GABA in the different fermented medium samples. Column temperature was set to 30 °C, sample temperature was set to 10 °C, and injection volume was 3 µL. An isocratic elution with a mixed in volume of 5% of acetonitrile (HPLC grade, Scharlab, Barcelona, Spain) and 95% of 0.1% formic acid (LC–MS grade, Scharlab,) prepared in Milli-Q water as mobile phase, and a flow rate of 0.25 mL/min, was used. A triple quadrupole MS equipped with an orthogonal electrospray ionisation (ESI) source (ACQUITY TQD, Waters) was used for detection. The instrument was operated in positive mode electrospray (ESI +), MS settings were used as follows: capillary voltage 3.05 kV, desolvation temperature 400 °C, source temperature 120 °C, cone and desolvation gas (nitrogen) flow 60 L/h and 800 L/h, respectively, and collision gas (argon) flow 0.10 mL/min. High purity nitrogen and argon were used (Nippon Gases, Madrid, Spain). MS was run in multiple reaction monitoring (MRM) including two ion transitions for GABA: m/z 104 > 87 for quantification and m/z 104 > 69 for identification. Data acquisition and quantification were performed using MassLynx software version 4.1 (Waters). Quantification was performed against a linear (1/x weighted) regression curve based on duplicate injections of calibration GABA standard solutions.

### Statistical analysis

The statistical analysis was carried out using the IBM-SPSS statistics software version 25.0 (IBM, New York USA). One-way analysis of variance (ANOVA) was used to evaluate the presence of statistically significant differences in the amount of GABA produced and the growth of *L. plantarum K16* strain among the fermented media within each fermentation parameter studied. Bonferroni's method was applied for pairwise comparison, and statistical significance was declared at *P* ≤ 0.05. In addition, Rho Spearman correlation coefficient was calculated to investigate the relationship between the amount of GABA produced and the nutritional composition of each agri-food by-product used.

## Results

### Effect of fermentation parameters in the production of GABA using MRS broth

#### Percentage of inoculum

Different initial inoculum percentage, 0.8% (7.41 ± 0.07 log CFU/mL), 1.0% (7.44 ± 0.06 log CFU/mL), 1.2% (7.50 ± 0.03 log CFU/mL) and 1.4% (7.60 ± 0.08 log CFU/mL), were assessed to determine the suitable concentration for producing the greatest GABA. The results, represented in Table 2, show that 977.03 ± 22.08 mg/L of GABA were produced when 0.8% of inoculum was added to the medium, and no significant difference (P > 0.05) was observed with respect to the amount of GABA produced with 1% of inoculum. Likewise, the microbial growth was not significantly different between both inoculum percentages after 96 h of fermentation (Table 2). Nevertheless, using a 1.2% inoculum, the GABA production significantly increased (P ≤ 0.05) to 1419.93 ± 57.47 mg/L, along with a pH of 4.30 ± 0.16 and a microbial growth of 7.31 ± 0.41 log CFU/mL. A higher inoculum, 1.4%, did not significantly (P > 0.05) increase the amount of GABA produced compared with the concentration reached with 1.2%. Consequently, an inoculum of 1.2% was selected to carry out the following experiments.

**Table 2:** GABA (mg/L), viable counts (log CFU/mL) and pH values obtained with *Lactiplantibacillus plantarum K16* in MRS broth, after 96 h of fermentation, using different percentages of inoculum, initial pH, MSG, and glucose concentration (Different letter superscripts indicate if the results are statistically significant (*P* ≤ 0.05) in the GABA, viable counts or pH values among the levels of each fermentation parameter).

	GABA (mg/L)	Viable counts (log CFU/mL)	pH
**Inoculum (percentage-log CFU/mL)**			
0.8–7.41	977.03 ± 22.08^*b*^	7.11 ± 0.03^*b*^	4.46 ± 0.03^*a*^
1.0–7.44	1000.23 ± 70.82^*b*^	6.99 ± 0.03^*b*^	4.42 ± 0.01^*a*^
1.2–7.5	1419.93 ± 57.47^*a*^	7.31 ± 0.14^*a*^	4.30 ± 0.16^*a*^
1.4–7.6	1428.27 ± 5.38^*a*^	6.83 ± 0.04^*b*^	4.42 ± 0.01^*a*^
**Initial pH**			
4.0	197.50 ± 11.92^*d*^	8.07 ± 0.01^*a*^	3.97 ± 0.02^*b*^
4.5	951.05 ± 49.26^*c*^	6.68 ± 0.03^*c*^	4.03 ± 0.01^*b*^
5.5	1419.93 ± 57.47^*a*^	7.31 ± 0.14^*b*^	4.30 ± 0.16^*a*^
6.0	1323.01 ± 72.08^*b*^	7.91 ± 0.03^*a*^	4.21 ± 0.01^*a*^
**MSG (mM)**			
100	174.17 ± 46.7^*d*^	6.90 ± 0.11^*b*^	3.64 ± 0.01^*b*^
300	1207.14 ± 60.38^*b*^	7.43 ± 0.05^*a*^	4.05 ± 0.02^*b*^
500	1419.93 ± 57.47^*a*^	7.31 ± 0.14^*a*^	4.30 ± 0.16^*a*^
550	1027.81 ± 38.21^*c*^	7.39 ± 0.04^*a*^	4.36 ± 0.02^*a*^
**Glucose (g/L)**			
20	896.4 ± 29.85^*d*^	7.37 ± 0.02^*a*^	4.43 ± 0.03^*a*^
23	1391.2 ± 64.84^*c*^	7.29 ± 0.03^*a*^	4.45 ± 0.01^*a*^
25	2115.7 ± 73.83^*a*^	7.40 ± 0.14^*a*^	4.43 ± 0.02^*a*^
27	1771.6 ± 63.61^*b*^	7.28 ± 0.02^*a*^	4.37 ± 0.02^*a*^

### Initial pH

Several initial pH, between 4.0 and 6.0, was studied, focusing on identifying the most suitable to enhance the GABA synthesis. In this case, after 96 h of fermentation, a concentration of 197.5 ± 11.92 mg/L of GABA and no changes in the pH medium were observed using an initial pH of 4.0 (Table 2). However, when the initial pH raised to 4.5, the GABA amount significantly increased (*P* ≤ 0.05) up to 951.05 ± 49.26 mg/L, together with a slight decrease in the media pH up to 4.0. Furthermore, a significant increase (*P* ≤ 0.05) in the amount of GABA was obtained when the initial pH was 5.5 reaching the maximum value of GABA produced (1419.93 ± 57.47 mg/L). Contrarily, when the initial pH raised to 6.0, a substantial decrease (*P* ≤ 0.05) in the amount of GABA (1323.01 ± 72.08 mg/L) was observed compared with the value observed when the initial pH was 5.5. At the same time, the increase of GABA concentration during 96 h of fermentation was accompanied by a decrease in the growth of *L. plantarum K16* strain, hitting the concentration of 7.31 ± 0.14 log CFU/mL when the initial pH was 5.5.

### Concentration of MSG

The increase of MSG concentration showed a significant improve (*P* ≤ 0.05) in the GABA yield by *L. plantarum K16* strain (Table 2). Specifically, an MSG concentration of 100 mM resulted in 174.17 ± 46.7 mg/L of GABA and a microbial growth of 6.90 ± 0.11 log CFU/mL, and the amount of GABA significantly increased (*P* ≤ 0.05) up to 1207.14 ± 60.38 mg/L when the concentration of MSG was 300 mM. The maximum GABA production (1419.93 ± 57.47 mg/L) was reached at 500 mM of MSG concentration, although a significant decrease (*P* ≤ 0.05) in the amount of GABA was observed at MSG concentration greater than 500 mM (1027.81 ± 38.21 mg/L). On the other hand, no significant variation (*P* > 0.05) in the microbial growth was observed when the MSG concentration was higher than 300 mM (Table 2).

### Concentration of glucose

Glucose concentrations from 20 to 27 g/L were used to test the impact of this sugar on GABA production by *L. plantarum K16* strain. In the media with 20 g/L of glucose the concentration of GABA was 896.4 ± 29.85 mg/L and the microbial cell growth was 7.37 ± 0.02 log CFU/mL (Table 2). A significant increase (*P* ≤ 0.05) of GABA synthesis (1391 ± 64.84 mg/L) was observed when the glucose concentration reached 23 g/L in the medium. The maximum concentration of GABA (2115.70 ± 73.83 mg/L) was observed with 25 g/L of glucose, but a higher concentration of glucose (27 g/L) resulted in a significant decrease (*P* ≤ 0.05) in the amount of GABA produced (1771.6 ± 63.61 mg/L) (Table 2). Regardless of the concentration of glucose supplied to the culture medium, the microbial cell growth did not significantly change (*P* > 0.05) maintaining viable counts around 7 log CFU/mL (Table 2). According with these results, 25 g/L of glucose supplementation was considered the optimal concentration to obtain the highest GABA amount during fermentation.

### GABA production using agri-food by-products

A production trial of GABA was performed using different kinds of agri-food by-products as fermentation substrates for *L. plantarum K16.* In this case, GABA synthesis was stimulated by applying the best conditions observed using MRS broth. Therefore, the GABA produced in MRS broth was considered the control and was used to compare the results observed in the fermented by-products. The results show that the fermentation of apple by-product yielded 1166.81 ± 27.46 mg/L of GABA and a microbial cell growth of 8.13 ± 0.04 log CFU/mL (Table 3). GABA production using orange by-product was quite similar (1280.01 ± 59.22 mg/L) to that of apple by-product but with a significant increase (*P* ≤ 0.05) in the microbial growth reaching a concentration of 8.88 ± 0.14 log CFU/mL. Green pepper and tomato by-products significantly (*P* ≤ 0.05) enhanced the biosynthetic pathway of GABA producing 1626.52 ± 55.9 mg/L and 1776.75 ± 109.49 mg/L, respectively (Table 3). However, the GABA yield of *L. plantarum K16* was significantly higher (2115.7 mg/L) compared to the values observed using agri-food by-products.

**Table 3:** Content of GABA (mg/L), viable counts (log CFU/mL) and pH values achieved with *L. plantarum K16* fermenting tomato, green pepper, apple, and orange by-products and MRS broth as a control (Different letter superscripts indicate if the results are statistically significant (*P* ≤ 0.05) in the GABA content, viable counts, or pH values among the agri-food by-products).

Agri-food by-product	GABA (mg/L)	Viable counts (log CFU/mL)	pH
Control (MRS broth)	2115.7 ± 73.83^*a*^	7.40 ± 0.14^*c*^	4.43 ± 0.02^*a*^
Tomato	1776.75 ± 109.49^*b*^	8.17 ± 0.02^*b*^	4.44 ± 0.01^*a*^
Green pepper	1626.52 ± 55.90^*b*^	7.69 ± 0.08^*c*^	4.46 ± 0.01^*a*^
Apple	1166.81 ± 27.46^*c*^	8.13 ± 0.04^*b*^	4.27 ± 0.01^*b*^
Orange	1280.01 ± 53.22^*c*^	8.88 ± 0.14^*a*^	4.29 ± 0.04^*b*^

## Discussion

The first aim of this research has focused on identifying fermentation parameters involved in GABA synthesis. Therefore, an OFAT experiment was carried out in MRS broth to evaluate how the percentage of inoculum, initial pH, MSG, and glucose concentration influence *L. plantarum K16* to produce GABA. When the percentage of inoculum was assayed, a significant increase in the amount of GABA was observed using an inoculum of 7.5 log CFU/mL (1.2%) compared to using an inoculum of 7.41 log CFU/mL (0.8%) (Table 2). However, an inoculum of 7.6 log CFU/mL (1.4%) did not significantly increase GABA yield. Other studies also reported the importance of the inoculum concentration to enhance the biosynthesis of GABA. For instance, Kantachote et al.^40^ showed that *L. plantarum DW12* produced the highest concentration of GABA (128 mg/L) when the initial inoculum was 7 log CFU/mL giving a microbial cell growth of 8.01 log CFU/mL. However, a higher inoculum (8 log CFU/mL) increased the microbial cell growth to 9.2 log CFU/mL but yielded 101 mg/L of GABA. Rayavarapu et al.^28^ showed that the highest amount of GABA produced by *L. fermentum* was 3.79 g/L and the microbial cell growth was 5.8 log CFU/mL using a 1% of inoculum. However, an increase of inoculum to 2% did not significantly change the GABA production yielding 3.71 g/L and a microbial cell growth of 6.4 log CUF/mL. Even lower GABA synthesis was observed when the inoculum used was 3 or 4% obtaining 2.62 and 2.12 g/L of GABA, respectively.

Regarding the initial pH of the culture medium, LAB are broadly adapted to a wide range of pH values mainly due to the acid stress caused by their metabolism, because LAB normally produce a wide amount of lactic acid from carbohydrates fermentation. A high concentration of lactic acid creates a stressful environment in the medium that could negatively influence bacterial development and cause growth inhibition, while nutrients are still available, as well as increase cell death^44^. Consequently, LAB have developed protective mechanisms to avoid cell damage^45^. In this sense, Heunis et al.^46^ identified about 300 proteins involved in the protection of *L. plantarum 423* strain against acid stress. Most of protective mechanisms try to maintain the intracellular pH using proton pumps, decarboxylation, deamination, metabolism changes, or strengthening the cell envelop. Fernández and Zúñiga^47^ highlighted the importance of the catabolism of amino acids, such as aspartic acid, arginine or glutamic acid, as critical coping mechanism to overcome stressful environments. The GAD pathway is considered one of the most essential acid tolerance systems, which is based on the decarboxylation of glutamic acid by a GAD enzyme resulting in a molecule of GABA, classified as an alkaline compound^48^. In addition, during GAD pathway, a cytoplasmic proton is consumed increasing the internal pH and improving cell homeostasis maintenance^49^. Shin et al.^50^ indicated that the catalytic activity of GAD enzyme is extremely dependent on pH, and the optimum pH value significantly enhance the relative activity of the enzyme and thus the GABA yield. In our study, the highest GABA production (Table 2) was detected when the initial pH was 5.5. Zhang et al.^51^ and Chen et al.^41^ also reported that other *L. plantarum* strains produced the highest amount of GABA in MRS broth when the initial pH of the medium was 5.5. Similarly, Tanamool et al.^52^ reported that an increase in the initial pH from 4.0 to 6.0 significantly increased the amount of GABA produced (from 2 to 14 g/L) by a *L. plantarum* strain isolated from fermented fish products.

Generally, LAB are considered nutritionally fastidious microorganisms, which need the supplementation of vitamins and amino acids required for a proper metabolism performance^53^. Hence, the development of *L. plantarum* strains could be linked to the supplementation of amino acids because, in many cases, these bacteria are unable to produce these compounds. For instance, *L. plantarum* could need l-Glu supplementation to metabolise it and enhance the bacteria growth^54^. Likewise, l-Glu could also be required to activate the secondary metabolism to produce postbiotic compounds such as GABA^55^ or plantaricin^56^. Furthermore, l-Glu is usually supplemented directly into the fermentation media of *L. plantarum* strains due to this amino acid is the GABA precursor^57^. In the same way, MSG has been used in several studies to enhance GABA synthesis^58–60^. However, the MSG concentration should be optimised for each strain due to an excessive MSG concentration could be toxic and suppress the GAD enzyme^55^. In this investigation, increasing the concentration of MSG from 100 to 500 mM significantly enhanced GABA synthesis, but a reduction in GABA production was observed by supplying 550 mM of MSG (Table 2). Harnentis et al.^26^ also reported that *L. plantarum N5,* isolated from buffalo milk, achieved the highest amount of GABA (18 g/L) using a glutamate concentration of 500 mM. However, since MSG concentration is strain-dependent, other studies performed with *L. plantarum* strains reported that 80 and 200 mM of MSG were optimal for GABA synthesis^61,62^. Similarly, Yogeswara et al.^58^ studied the GABA production of *L. plantarum FNCC 260* strain using a wide range of MSG concentrations. The results showed a maximum GABA production (1226 mg/L) by supplying to MRS broth with 100 mM of MSG. Gomaa^63^ required a concentration of 750 mM MSG to get the maximum GABA yield (14.5 g/L) using *L. plantarum DSM749* strain isolated from Egyptian dairy products. Among other LAB species, the optimum amount of MSG can be also highly variable. Villegas et al.^64^ studied the GABA production using an *L. brevis* strain isolated from quinoa sourdough. MRS medium was supplied with concentrations of MSG up to 400 mM, reaching the highest concentration of GABA (26.29 g/L) with 270 mM of MSG. Likewise, Wu et al.^30^ increased the efficiency of the GABA synthesis by *L. brevis RK03* strain reaching 62.53 mg/L of GABA by supplying 650 mM of MSG to the fermented medium.

The source of sugar is also essential for LAB species to produce energy and cell biomass^65^. In this regard, glucose is considered the most attractive carbohydrate commonly used to enhance bacterial cell growth and lactic acid production^66,67^. Moreover, glucose catabolism produces severe acidification of the medium that could trigger the activation of the GAD pathway and thus, the stimulation of GABA synthesis^68^. In the present study, when the MRS broth contained 25 g/L of glucose, *L. plantarum K16* synthetised the great concentration of 2115.7 mg/L of GABA. Furthermore, Hussin et al.^69^ reported the highest GABA synthesis by *L. plantarum Taj-Apis362* using 20 g/L of glucose. However, *L. plantarum EJ2014* only required 10 g/L of glucose to yield 19.8 g/L of GABA^70^ and *L. plantarum KCTC3103* showed the maximum GABA production (670 mg/L) using 5 g/L of glucose^71^. Contrary, Zareian et al.^72^ using *L. plantarum MNZ* strain isolated from fermented soybean showed the highest GABA (408.36 mg/L) biosynthesis when 60 g/L of glucose were supplied to the fermentation media.

The fermentation process in MRS broth helped identify the essential parameters to produce GABA by *L. plantarum K16.* The maximum concentration of GABA (2115.7 mg/L) was obtained using MRS broth composed of 25 g/L of glucose, 12 g/L of yeast extract, 500 mM of MSG, an initial pH of 5.5, an inoculum of 1.2%, incubated at 34 °C and fermented for 96 h. Therefore, after identifying the best conditions to produce the maximum amount of GABA by *L. plantarum K16* in MRS broth, a fermentation trial was performed to assess the ability of this bacteria to produce GABA in agri-food by-products. According to the nutritional and functional value of orange, green pepper, tomato, and apple, their pulp and seeds by-products were considered suitable raw materials for fermentation. Several authors^73,74^ have proposed recycling apple waste by using it as a fermentation substrate due to its high concentration of magnesium, calcium, fibre, and phenolic compounds like flavonoids or hydroxycinnamic acid. Moreover, more than half of the raw material from the orange juice industry are wasted, which means the loss of a good source of dietary fibre, phenolic compounds, and minerals^75,76^. Likewise, pepper and tomato by-products are also considered good sources of dietary fibre, phenolic compounds, proteins, carbohydrates, and lipids^77,78^. Likewise, Table 1 shows that apple by-products had the highest concentration of total carbohydrates and sugars, followed by orange, green pepper, and tomato. However, the protein content in apple by-product was the lowest compared with that of tomato by-product. Furthermore, the tomato by-product reported the highest concentration of l-Glu (335 mg/100 g), followed by green pepper, orange, and apple by-product. Despite the nutritional variability between these four by-products, in the present study, they were enriched with 25 g/L of glucose, 12 g/L of yeast extract and 500 mM of MSG, to ensure that at least *L. plantarum K16* had enough nutrients to synthetise GABA. Furthermore, *L. plantarum K16* produced great amount of GABA reaching a concentration of 1166.81 mg/L, 1280.01 mg/L, 1626.52 mg/L and 1776.75 mg/L in apple, orange, green, and tomato by-products, respectively (Table 3). However, the GABA produced using MRS broth was significantly higher than the concentration obtained with any of the agri-food by-products. Sharma et al.^79^ also evaluated if *L. plantarum LP-9* could produce GABA using saccharified agro-residues such as wheat rice, corn bran or cassava. In this case, they also performed a previous optimisation process, in MRS broth, of relevant parameters for GABA production, such as MSG, pH, and temperature. Then, these optimised parameters were applied in those agri-residues showing the maximum production of GABA (1.39 g/L) using cassava, but it was lower concentration than the one observed in MRS broth (1.53 g/L). Contrarily, Moo-Chang et al.^80^ showed that *L. sakei B2-16* in MRS broth enriched with 4% of sucrose, 1% of yeast extract and 5% of MSG (conditions previously optimised) could produce 28.05 g/L of GABA. However, significantly higher concentration of GABA, 68.05 g/L, was obtained using the by-product rice bran extract enriched with 4% of sucrose, 1%yeast extract and 12% of MSG.

In the present study, the difference in GABA yield between each agri-food by-product could be related to the variability in their nutritional composition. Regarding Table 1, carbohydrate and sugar concentrations of the agri-food by-products were inversely correlated ( ≥|0.6|) with GABA production. However, the microbial cell growth showed a positive correlation (≥ 0.4) with carbohydrate and sugar content. On the other hand, it was observed a strong direct correlation (≥ 0.8) between the content of GABA and protein, as well as the concentration of l-Glu (≥ 0.9). This could mean that agri-food by-products with high content of sugar and carbohydrates could enhance metabolic pathways involved in cell duplication. Nevertheless, a higher protein and l-Glu concentration could enhance the GAD pathway.

Furthermore, the different production of GABA, between MRS broth and by-products, could be due to agri-food by-products present a wide and great variety of compounds compared to MRS broth, which composition is fully controlled. Thus, the variability of compounds in each agri-food by-product could have different effects on *L. plantarum K16* metabolism. For example, several compounds could activate other metabolic pathways on *L. plantarum K16* strain by focusing more on these biochemical processes than on the GAD pathway. Several studies have reported the importance of other metabolic routes that protect LAB under stressful conditions such as arginine or agmatine deaminase pathways or aspartic acid or histidine decarboxylation processes^44,47–82^. Therefore, after confirmed that tomato, orange, apple, and green pepper by-products could be used to produce GABA by *L. plantarum K16*. Further research is necessary to characterise the composition of each by-product and design a specific optimisation process for each by-product to maximise the GABA production of *L. plantarum K16.*

## Conclusions

A wide range of relevant parameters involved in the GABA production were individually studied to achieve the highest yield of *L. plantarum K16* strain*.* The optimisation of the percentage of inoculum, the initial pH, MSG, and glucose concentration, strongly influenced the GAD pathway of *L. plantarum K16* and significantly increased the GABA production in MRS broth. Afterwards, GABA production was successfully achieved using tomato, green pepper, apple, and orange by-products by applying previously optimised fermentation parameters.

## Data Availability

All the data generated in the study are included in the present manuscript. All the materials described are available from the corresponding author upon reasonable request.
